# Local application of IGFBP5 protein enhanced periodontal tissue regeneration via increasing the migration, cell proliferation and osteo/dentinogenic differentiation of mesenchymal stem cells in an inflammatory niche

**DOI:** 10.1186/s13287-017-0663-6

**Published:** 2017-09-29

**Authors:** Nannan Han, Fengqiu Zhang, Guoqing Li, Xiuli Zhang, Xiao Lin, Haoqing Yang, Lijun Wang, Yangyang Cao, Juan Du, Zhipeng Fan

**Affiliations:** 1 Laboratory of Molecular Signaling and Stem Cells Therapy, Beijing Key Laboratory of Tooth Regeneration and Function Reconstruction, Capital Medical University School of Stomatology, Beijing, 100050 China; 2 Department of Periodontology, Capital Medical University School of Stomatology, Beijing, 100050 China; 3 Molecular Laboratory for Gene Therapy and Tooth Regeneration, Beijing Key Laboratory of Tooth Regeneration and Function Reconstruction, Capital Medical University School of Stomatology, Beijing, 100050 China; 4 Department of Implant Dentistry, Capital Medical University School of Stomatology, Beijing, China; 5 Department of Endodontics, Capital Medical University School of Stomatology, Beijing, China

**Keywords:** Insulin-like growth factor binding protein 5 (IGFBP5), Mesenchymal stem cells (MSCs), Periodontal tissue regeneration, BCOR, KDM6B

## Abstract

**Background:**

Periodontitis is a widespread infectious disease ultimately resulting in tooth loss. The number of mesenchymal stem cells (MSCs) in patients with periodontitis is decreased, and MSC functions are impaired. Rescuing the impaired function of MSCs in periodontitis is the key for treatment, especially in a manner independent of exogenous MSCs. Our previous study found that overexpressed insulin-like growth factor binding protein 5 (IGFBP5) could promote exogenous MSC-mediated periodontal tissue regeneration. Here, we investigate the role of IGFBP5 protein in MSCs and periodontal tissue regeneration independent of exogenous MSCs in an inflammatory niche.

**Methods:**

TNFα was used to mimic the inflammatory niche. Lentiviral *IGFBP5* shRNA was used to silence *IGFBP5* and recombinant human IGFBP5 protein (rhIGFBP5) was used to stimulate the periodontal ligament stem cells (PDLSCs) and bone marrow stem cells (BMSCs). The effects of IGFBP5 on PDLSCs were evaluated using the scratch-simulated wound migration, Transwell chemotaxis, alkaline phosphatase (ALP) activity, Alizarin red staining, Cell Counting Kit-8, Western blot, Real-time PCR, Co-IP and ChIP assays. The swine model of periodontitis was used to investigate the functions of IGFBP5 for periodontal regeneration and its anti-inflammation effect.

**Results:**

We discovered that 0.5 ng/ml rhIGFBP5 protein enhanced the migration, chemotaxis, osteo/dentinogenic differentiation and cell proliferation of MSCs under the inflammatory condition. Moreover, 0.5 ng/ml rhIGFBP5 application could rescue the impaired functions of *IGFBP5*-silenced-MSCs in the inflammatory niche. Furthermore, local injection of rhIGFBP5 could promote periodontal tissue regeneration and relieve the local inflammation in a minipig model of periodontitis. Mechanistically, we found that *BCOR* negatively regulated the expression of *IGFBP5* in MSCs. BCOR formed a protein complex with histone demethylase KDM6B and raised histone K27 methylation in the *IGFBP5* promoter.

**Conclusions:**

This study revealed that rhIGFBP5 could activate the functions of MSCs in an inflammatory niche, provided insight into the mechanism underlying the activated capacities of MSCs, and identified IGFBP5 as a potential cytokine for improving tissue regeneration and periodontitis treatment independent of exogenous MSCs and its potential application in dental clinic.

**Electronic supplementary material:**

The online version of this article (doi:10.1186/s13287-017-0663-6) contains supplementary material, which is available to authorized users.

## Background

Periodontitis is a chronic inflammatory disease of the periodontal tissues and is characterized by periodontal-supported tissue loss, including the absorption of alveolar bone, ultimately leading to tooth loss [1]. It is well known that chronic periodontitis is caused by infectious bacteria and host response [2, 3]. At present, periodontitis treatment involves conventional methods such as scaling, root planing and guided tissue regeneration; however, these do not achieve ideal periodontal tissue regeneration and inflammation control [4, 5]. Therefore, periodontal tissue regeneration and inflammation control are the key issues for periodontitis treatment. Currently, mesenchymal stem cell (MSC)-mediated periodontal tissue regeneration is regarded as a hopeful method for periodontitis treatment, specifically as a periodontal tissue engineering technique used to treat periodontitis. MSCs are characterized by multilineage differentiation and self-renewal properties and are identified as reliable seed cells for tissue regeneration [6–8]. It has been demonstrated that transplantation of autologous or allogeneic MSCs into periodontal lesion areas can promote periodontal tissue regeneration [9–12]. Although the transplantation of MSCs, as a promising treatment for periodontal tissue regeneration, has made remarkable strides in recent years, some key problems still exist, such as the unclear regulation mechanism of MSCs, security and ethics, etc. In addition, there are several other approaches for periodontal tissue regeneration, including utilization of an enamel matrix derivative (EMD) and a variety of growth factors, such as transforming growth factor-β, insulin-like growth factor 1 (IGF-1) and platelet-rich plasma (PRP) [13–15]. However, recent results have shown that it is difficult to attain the desired tissue regeneration. The main problem is that the number of periodontal ligament stem cells (PDLSCs) in patients with periodontitis is decreased, and their functions are impaired, which impedes the regeneration of periodontal tissues [16–18]. Thus, enhancing the endogenous functions of MSCs in periodontitis is the key for treatment, especially in a manner independent of exogenous MSCs.

Insulin-like growth factors (IGFs) and their binding proteins (IGFBP1–6) play a crucial role in the function of osteoblasts and bone formation in vivo and in vitro [19, 20]. Insulin-like growth factor binding protein 5 (IGFBP5) is one member of this binding protein family that has been shown to enhance cell growth, remodelling and repair of bone. Moreover, research has discovered that daily subcutaneous injections of recombinant IGFBP5 protein stimulates osteoblast activity and bone accretion in ovariectomized mice [21–23]. Our previous study also found that IGFBP5 expression was significantly decreased in PDLSCs and gingival crevicular fluid of periodontitis patients, and overexpressed *IGFBP5* could promote exogenous MSC-mediated periodontal tissue regeneration via enhancing osteo/dentinogenic differentiation and the anti-inflammation capacities of MSCs. With regard to mechanism, we demonstrated that *IGFBP5* was a downstream target gene of lysine (K)-specific demethylase 6B (KDM6B) and that KDM6B promoted *IGFBP5* transcription by decreasing histone K27 methylation in the *IGFBP5* promoter [24]. However, the function of IGFBP5 protein in the regulation of MSCs in an inflammatory niche and whether it could promote periodontal tissue regeneration in periodontitis, especially independent of exogenous MSCs, is still not clear.

In this study, we investigated the role of IGFBP5 protein in the regulation of MSC function and periodontal tissue regeneration independent of exogenous MSCs in an inflammatory niche. Our results revealed that recombinant human IGFBP5 protein (rhIGFBP5) could activate the migration, chemotaxis, osteo/dentinogenic differentiation and cell proliferation of PDLSCs and bone marrow stem cells (BMSCs) in an inflammatory niche. Additionally, the local injection of rhIGFBP5 restored tissue lesions in periodontitis and had an anti-inflammatory effect in a minipig model of periodontitis. Our results identified a potential cytokine, IGFBP5, for improving tissue regeneration and periodontitis treatment in a manner independent of exogenous MSCs.

## Methods

### Cell cultures

Human stem cell research abided by the ISSCR “Guidelines for the Conduct of Human Embryonic Stem Cell Research.” Human impacted third molar teeth were obtained with informed patient agreement and following the rules approved by the Beijing Stomatological Hospital, Capital Medical University (Ethics Committee Agreement, Beijing Stomatological Hospital Ethics Review No. 2011-02). Solutions of 75% ethanol and phosphate-buffered saline (PBS) were used to disinfect and wash the teeth. PDLSCs were isolated, cultivated, and recognized as previously depicted [8–10]. Briefly, periodontal tissues were isolated from the periodontal ligament in the middle one-third of the tooth root. A solution of 3 mg/ml collagenase type I (Worthington Biochemical Corp, Lakewood, NJ, USA) and 4 mg/ml dispase (Roche Diagnostics Corp., Indianapolis, IN, USA) were utilized to digest the tissues for 1 h at 37 °C. Single PDLSCs suspensions were obtained by cell passage using a 70-μm strainer (Falcon, BD Labware, Franklin Lakes, NJ, USA). Human BMSCs were purchased from ScienCell Research Laboratories (Carlsbad, CA, USA). MSCs were cultivated in a humidified incubator under 5% CO_2_ at 37 °C in DMEM alpha modified Eagle’s medium (Invitrogen, Carlsbad, CA, USA), with 15% fetal bovine serum (FBS; Invitrogen), 100 μg/ml streptomycin, 100 U/ml penicillin, and 2 mmol/l glutamine (Invitrogen). The culture medium was converted every 3 days. Tumor necrosis factor alpha (TNFα) (Peprotech, Rocky Hill, NJ, USA) and rhIGFBP5 (R&D Systems, Minneapolis, MN, USA) were used to treat PDLSCs.

### Plasmid construction and viral infection

The plasmids were constructed according to standard techniques, and all structures were testified by proper enzyme digestion and/or sequencing. Human full-length BCL6 co-repressor (*BCOR*) cDNA from PDLSCs compounded to an HA tag was manufactured with a standard PCR agreement. This sequence (*HA*-*BCOR*) was put into the PQCXIN retroviral vector via AgeI and BamH1 restriction sites. Short hairpin RNAs (shRNA) targeted the complementary sequences of the genes were inserted in the pLKO.1 lentiviral vector (Addgene, Cambridge, MA, USA). Viral packaging was prepared following the manufacturer’s protocol (Clontech Laboratories, Mountain View, CA, USA or Addgene). For viral infections, the cells were plated and infected with retroviruses or lentiviruses supplied with the polybrene (6 μg/ml, Sigma-Aldrich, St. Louis, MO, USA) for 12 h. After 48 hours, infected cells were selected with different antibiotics. A scrambled shRNA (Scramsh) was obtained from the company Addgene. The target sequences for the shRNA were: *IGFBP5* shRNA (*IGFBP5*sh), 5’-GCAGATCTGTGAATATGAA-3’; *BCOR* shRNA (*BCOR*sh), 5’-GATGGCTTCAGTGCTATAT-3’.

### Real-time reverse transcriptase-polymerase chain reaction (real-time RT-PCR)

Total RNA was secluded from MSCs by using Trizol reagents (Invitrogen). From 2-μg RNA aliquots, we integrated cDNA by oligo (dT) and reverse transcriptase, according to the manufacturer’s protocol (Invitrogen). Then, Real-time RT-PCR reactions were performed according to the QuantiTect SYBR Green PCR kit (Qiagen, Hilden, Germany) and an Icycler iQ Multi-color Real-time RT-PCR Detection System. The primers for specific genes are listed in Additional file 1: Table S1.

### Scratch migration assays

The scratch-simulated wound migration assay was implemented to assess the function of MSC migration. Upon 80% confluence, cells were digested using 0.25% trypsin-ethylene diamine tetraacetic acid (EDTA) (Gibco®, Life Technologies™, Carlsbad, CA, USA) and seeded onto six-well plates at a density of 2 × 10^5^ cells/well and allowed to grow close to 95% confluence. Cells were grown in DMEM alpha modified Eagle’s medium (Invitrogen, Carlsbad, CA, USA) without fetal bovine serum (FBS; Invitrogen). After culturing for 24 hours, a cross scratch was performed in the cell layer along the diameter of the well with a 200 μl pipet tip (Axygen® Corning, NY, USA) and the cells were grown in fresh culture media with 10 ng/ml TNFα. Images from the same view were taken under microscopy at baseline (0 h), 24 hours and 48 hours after wounding for determination of the extent of wound closure. Image-Pro 1.49v (National Institutes of Health, Bethesda, MD, USA) was used to measure the void area (VA) and the height and the relative width were evaluated (Area% = VA/height) in each group.

### Transwell chemotaxis assays

MSCs were cultured in Transwell chambers with an 8-μm pore size membrane (Corning, Costar, MA, USA). The upper chamber was seeded with MSCs (2.0 × 10^4^ cells) which were grown in 100 uL DMEM alpha modified Eagle’s medium (Invitrogen, Carlsbad, CA, USA) supplemented with TNFα (final concentration is 10 ng/ml) [25–27] and no fetal bovine serum (FBS; Invitrogen) while there was 600 uL DMEM alpha modified Eagle’s medium with TNFα (10 ng/ml) and 15% FBS in the bottom chamber. After 48 hours, the transferred cell numbers were counted in randomly selected fields using microscopy (OLYMPUS, Japan) at 200× magnification.

### Alkaline phosphatase (ALP) and Alizarin Red detection

MSCs were cultured in mineralization-inducing medium according to the StemPro osteogenesis differentiation kit (Invitrogen, Carlsbad, CA, USA). ALP activity was evaluated with an ALP capacity kit following the manufacturer’s protocol (Sigma-Aldrich, St. Louis, MO, USA). Signals were standardized based on protein concentrations. For confirming mineralization, cells were induced for 3 weeks, 70% ethanol was used to fix the cells, and 2% Alizarin red was used to stain the cultures (Sigma-Aldrich, St. Louis, MO, USA).

### Cell Counting Kit-8 (CCK8) assay

The assay was performed using a Cell Counting Kit-8 kit (Dojindo, Kumamoto, Japan) following the manufacturer’s protocol. A volume of 100 uL of MSCs suspension (5000 cells/well) was dispensed into a 96-well plate. TNFα (10 ng/ml) was used to treat the MSCs. The plate was pre-incubated for 48 hours in a humidified incubator (37 °C,5% CO2), 10 uL of various concentrations of toxicant was added into the culture media in the plate, and then the plate was incubated in the incubator for 1-4 hours. The absorbance (OD) was measured at 450 nm using a microplate reader.

### Co-immunoprecipitation (Co-IP) assay

The assay was performed using a HA-Tag Co-IP kit (Thermo Fisher Scientific, Waltham, MA, USA) according to the manufacturer’s protocol. Cultured cells were washed carefully with pre-chilled PBS two times; then, cold RIPA lysis buffer was added. Cell lysate was centrifuged at 14,000 × x g at 4 °C for 15 minutes and the supernatant transferred to new tubes immediately. A volume of 10 μL anti-HA agarose slurry was dispensed into each labeled spin column utilizing a wide-bore pipette tip. Gentle end-over-end mixing was performed and the solution incubated at 4 °C for at least 1 hour. A total of 0.5 mL of TBS-T was added to each column and the column gently converted with the collection tube two to three times and centrifuged for 10 seconds. The wash was rendered up (or collected for future analysis). Elution Buffer (10 μL) was added to the anti-c-HA agarose, the tube gently tapped to mix and centrifuged for 10 seconds. For decreasing gel analysis, reducing sample buffer was prepared by adding 10 μL of 1 M DTT or 5 μL of 2-mercaptoethanol to the 40 μL of Lane Marker Non-Reducing Sample Buffer (5×) and 7.5 μL of the above-described reducing sample buffer added to 30 μL of elution sample. The sample was heated for 10 minutes at 95–100 °C and the supernatant collected for Western blot analysis.

### Western blot analysis

RIPA buffer (1 mM EDTA, 10 mM Tris-HCl, 1% sodium dodecyl sulphate [SDS], 1% NP-40, 50 mM β-glycerophosphate, 1:100 proteinase inhibitor cocktail, 50 mM sodium fluoride) was used to lyse cells. A 15% SDS polyacrylamide gel was used to isolate the samples which were then transferred to polyvinylidene difluoride (PVDF) membranes by using a semi-dry transfer apparatus (BioRad, Hercules, CA, USA). The membranes were blocked using 5% dehydrated milk for 1 h and then incubated with primary antibodies overnight. Horseradish peroxidase-conjugated anti-mouse or anti-rabbit IgG (Promega, Madison, WI, USA) were utilized to detect the immune compounds and samples viewed with SuperSignal reagents (Pierce, Rockford, IL, USA). Primary antibodies were obtained from commercial sources: mouse monoclonal anti-HA (Clone No.C29F4, Cat No.MMS-101P, Covance, Princeton, NJ, USA), polyclonal antibody against KDM6B (Merck Millipore, Billerica, MA, USA); monoclonal antibody against beta-actin (Applygen Technologies, Beijing. China).

### ChIP assays

The assay was performed using a ChIP assay detection kit (Merck Millipore, Darmstadt, Germany) according to the manufacturer’s protocol. Briefly, cells were cultivated with 1% formaldehyde for 10 minutes at 37 °C. Each reaction of ChIP was implemented using 2.0 × 10^6^ cells. For precipitation of DNA, 2 μg polyclonal antibodies against trimethyl H3K27 (H3K27me3; Cat No. ABE44, Merck Millipore, Darmstadt, Germany) were added. The DNA samples which precipitated were measured by real-time PCR using primers targeting the *HA*-*BCOR*-binding area of the *IGFBP5* promoter: forward, 5′-tacgtctcccttcagcctgt-3′; reverse, 5′-gagcagggtgaacacaatga-3′ [24]. Quantification data are represented as the percentage of input DNA.

### Animals

Nine inbred male minipigs (18–24 months old, weighing 50–55 kg) were obtained from the Institute of Animal Science of the Chinese Agriculture University (Beijing, China). Minipigs were raised under the conditions of free access to water and a regular provision of a soft food diet. The study agreement was ratified following the Animal Care and Use Committee of Capital Medical University. Before the surgery, the minipigs were clinically evaluated and then anesthetized with a combination of ketamine chloride (6 mg/kg) and xylazine (0.6 mg/kg) injected intramuscularly.

### rhIGFBP5 application in swine periodontitis model

A total of 12 experimental periodontitis defects were established in the six minipigs. Alveolar bone was cut to generate lesion areas with a size of 3 mm × 5 mm × 7 mm in the mesial region of the mandibular first molars. Then, after that, 4–0 silk ligament was sutured around the cervical portion of the first molars. Three minipigs without experimental periodontitis were used as healthy controls. Animals with experimental periodontitis were randomly assigned to two groups: injection of 0.9% NaCl (NaCl group), or injection of 0.5 ng/ml rhIGFBP5 (rhIGFBP5 group). Each group contained six defects in three minipigs. Postoperative injections were received by minipigs at three areas around each defect: the distal of the molar, the mesial of the molar, and the middle of the molar. Each injection needle was inserted from the mucosa to the surface of the bone (supra periosteal), confronting significant resisting force before the 0.9% NaCl or rhIGFBP5 was injected. For rhIGFBP5 treatment, each area surrounding the defect was injected with 10 uL of 0.5 ng/ml rhIGFBP5, and injection occurred once every 2 weeks.

### Clinical assessments of periodontal tissue regeneration

Clinical assessments, including gingival recession (GR), probing depth (PD), and attachment loss (AL), were measured on all experimental area pre-injection and post-injection at 4 weeks and 12 weeks. We used a Williams periodontal probe (Shanghai Kangqiao Dental Instruments Factory, Shanghai, China) to measure PD values. Bone regeneration was calculated by three-dimensional reconstructive computed tomography (CT) scan (Siemens, Berlin, Germany). The scanning length of the CT was 0.62 mm.

### Imaging and histological assessments

For assessment of periodontal tissue regeneration, all animals were sacrificed with CO_2_, and the samples of mandibular bone were dissected and fixed with 10% formaldehyde. After 2 weeks, all samples were decalcified with buffered 50% formic acid and imbedded in paraffin. The sections were deparaffinized and stained with hematoxylin and eosin. For histopathological judgement, buccal-lingual-direction parts of the experimental areas were dissected. The images were taken in experimental fields using microscopy (Olympus, Tokyo, Japan) at × 12.5, ×200 and × 400 magnification. Image-Pro 1.49v (National Institutes of Health, Bethesda, MD, USA) was used to measure the width and the height of new cementum (n = 6).

### Statistics

All statistical calculations were implemented by SPSS10 statistical software (SPSS Inc., Chicago, IL, USA) The Student’s *t* test, or one-way ANOVA were used to identify statistical significance, with a *P* ≤ 0.05 considered significant.

## Results

### Depletion of *IGFBP5* inhibited the functions of PDLSCs in the inflammatory condition

**Fig. 1:**
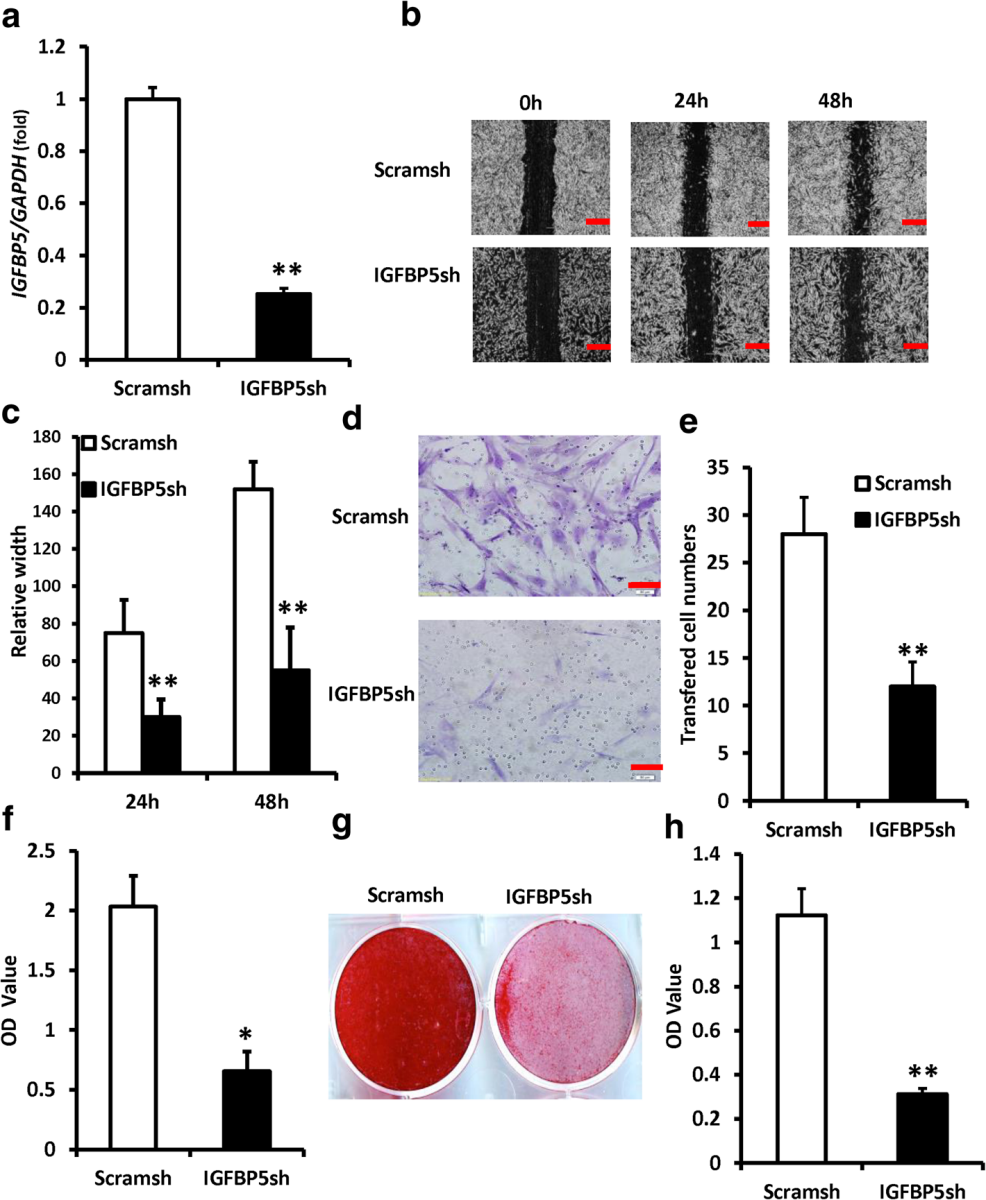
Depletion of *IGFBP5* inhibited the functions of PDLSCs in inflammatory condition. TNFα (10 ng/ml) was used to treat PDLSCs. **a** Real-time PCR results. *GAPDH* was used as an internal control. Short hairpin RNAs were utilized to infect PDLSCs and to silence *IGFBP5* (*IGFBP5*sh) or scramble shRNA (Scramsh). **b**, **c** The scratch-simulated wound migration assay results. Scale bar: 100 μm. **d**, **e** The Transwell chemotaxis assay results. Scale bar: 50 μm. **f** ALP activity. **g** Alizarin red staining. **h** Cell Counting Kit-8 assay results. Student’s *t* test was implemented to test statistical significance. Error bars represent SD (n = 3). *P ≤ 0.05; **P ≤ 0.01. *IGFBP5* insulin-like growth factor binding protein 5

*IGFBP5* was silenced using Lentiviral *IGFBP5* shRNA in periodontal ligament stem cells (PDLSCs), the knockdown efficiency was testified by real-time RT-PCR after selection with puromycin (Fig. 1a), and 10 ng/ml tumor necrosis factor alpha (TNFα) was used to mimic the inflammatory condition. The scratch-simulated wound migration assay results showed that *IGFBP5* knockdown inhibited the migration ability of PDLSCs at 24 and 48 hours after TNFα treatment (Fig. 1b, c). The Transwell chemotaxis assay results showed that *IGFBP5* knockdown inhibited the chemotaxic ability of PDLSCs at 48 hours under TNFα treatment (Fig. 1d, e). To evaluate the osteogenic differentiation abilities, transduced PDLSCs were cultivated in osteogenic-inducing medium. Five days after osteogenic induction, the knockdown of *IGFBP5* reduced ALP activity in PDLSCS compared to Scramsh-infected cells under TNFα treatment (Fig. 1f). Three weeks after osteogenic induction, *IGFBP5* knockdown inhibited mineralization in PDLSCs under TNFα treatment, as described by the results of Alizarin red staining (ARS) (Fig. 1g). To test whether depletion of *IGFBP5* causes PDLSCs cell proliferation changes, the Cell Counting Kit-8 was used to measure cell proliferation in PDLSCs. The results showed that *IGFBP5* knockdown inhibited the cell proliferation capacity of PDLSCs at 48 hours under TNFα treatment (Fig. 1h).

### rhIGFBP5 enhanced the functions of PDLSCs and BMSCs in the inflammatory condition

**Fig. 2:**
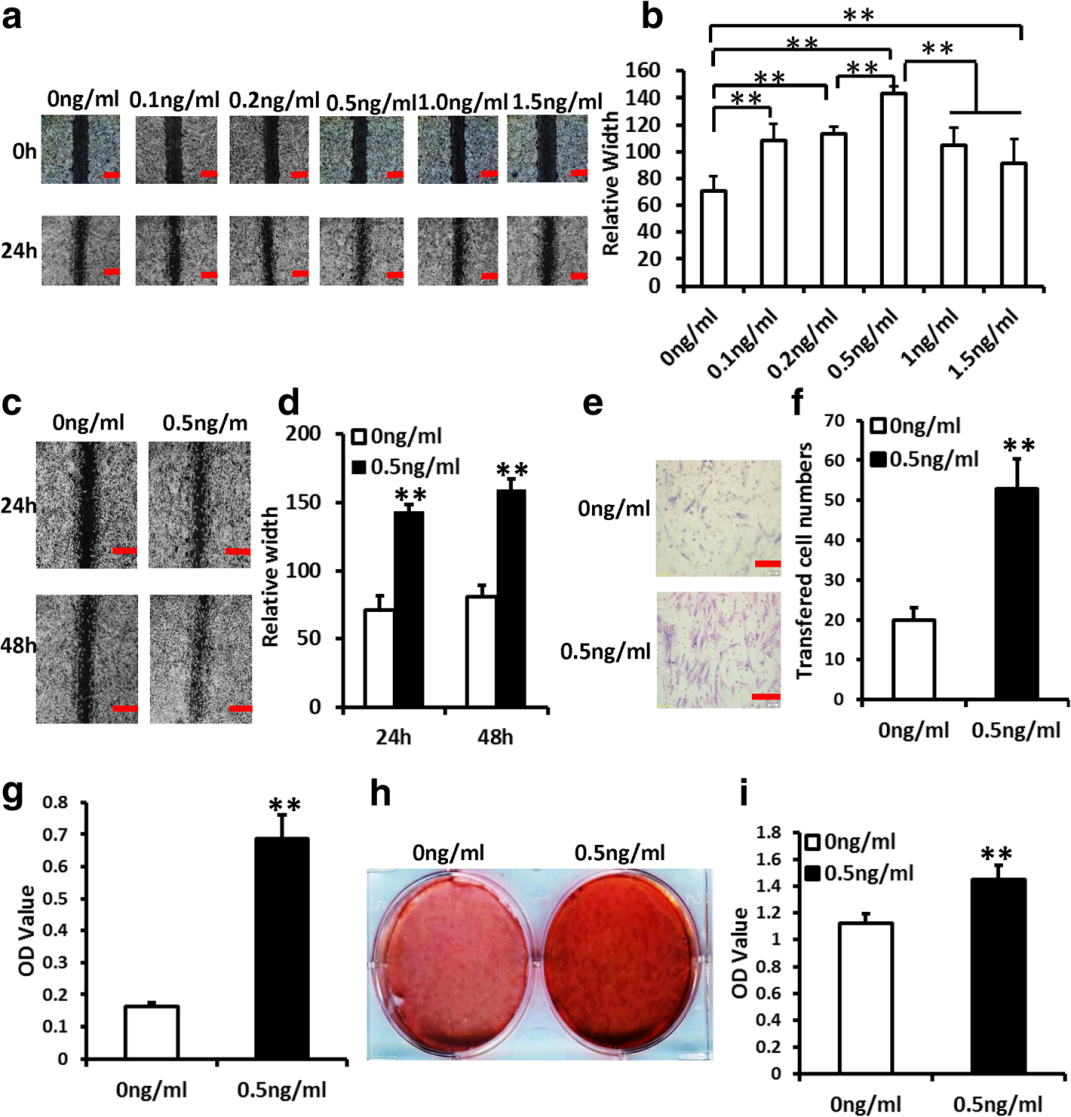
rhIGFBP5 enhanced the functions of PDLSCs in the inflammatory condition. TNFα (10 ng/ml) was used to treat PDLSCs. **a**-**d** The scratch-simulated wound migration assay results. Scale bar: 100 μm. **e**, **f** The Transwell chemotaxis assay results. Scale bar: 50 μm. **g** ALP activity. **h** Alizarin red staining. **i** Cell Counting Kit-8 assay results. One-way ANOVA was utilized for analysis in a-b. Student’s *t* test was utilized for analysis in **c**-**i**. Error bars represent SD (n = 3). **P ≤ 0.01

To detect the effect of rhIGFBP5 on MSCs in an inflammatory niche, 10 ng/ml TNFα was used to mimic the inflammatory condition. According to scratch-simulated wound migration assay results, we discovered that 0.5 ng/ml was the optimal concentration for rhIGFBP5 promotion of PDLSC migration at 24 hours under TNFα treatment (Fig. 2a, b). In addition, scratch-simulated wound migration assay results further confirmed that 0.5 ng/ml rhIGFBP5 could increase cell migration ability at 24 and 48 hours under TNFα treatment (Fig. 2c, d). The Transwell chemotaxis assay results showed that 0.5 ng/ml rhIGFBP5 enhanced the chemotaxic ability of PDLSCs at 48 hours under TNFα treatment (Fig. 2e, f). Next, 5 days after osteogenic induction, ALP activity assay results indicated that 0.5 ng/ml rhIGFBP5 increased the ALP activity in PDLSCs under 10 ng/ml TNFα treatment (Fig. 2g). Three weeks after osteogenic induction, 0.5 ng/ml rhIGFBP5 significantly enhanced mineralization in PDLSCs under TNFα treatment, as determined by ARS (Fig. 2h). The Cell Counting Kit-8 assay results showed that 0.5 ng/ml rhIGFBP5 markedly promoted cell proliferation in PDLSCs compared to the control group under TNFα treatment (Fig. 2i).

To verify whether rhIGFBP5 had similar abilities in other MSCs, BMSCs were treated with 10 ng/ml TNFα and 0.5 ng/ml rhIGFBP5. The scratch-simulated wound migration assay results indicated that 0.5 ng/ml rhIGFBP5 promoted migration ability in BMMSCs under TNFα treatment (Additional file 2: Figure S1a, b). The Transwell chemotaxis assay results showed that 0.5 ng/ml rhIGFBP5 promoted the chemotaxic ability of BMSCs under TNFα treatment (Additional file 2: Figure S1c, d). Further, 0.5 ng/ml rhIGFBP5 enhanced ALP activity and mineralization in BMSCs under TNFα treatment, as determined by ALP activity assay and ARS (Additional file 2: Figure S1e, f). The Cell Counting Kit-8 assay results showed that 0.5 ng/ml rhIGFBP5 accelerated cell proliferation in BMSCs under TNFα treatment (Additional file 2: Figure S1g). These findings elucidated that 0.5 ng/ml rhIGFBP5 considerably enhanced the function of MSCs.

### rhIGFBP5 rescued the impaired functions of *IGFBP5*-silenced PDLSCs in the inflammatory condition

**Fig. 3:**
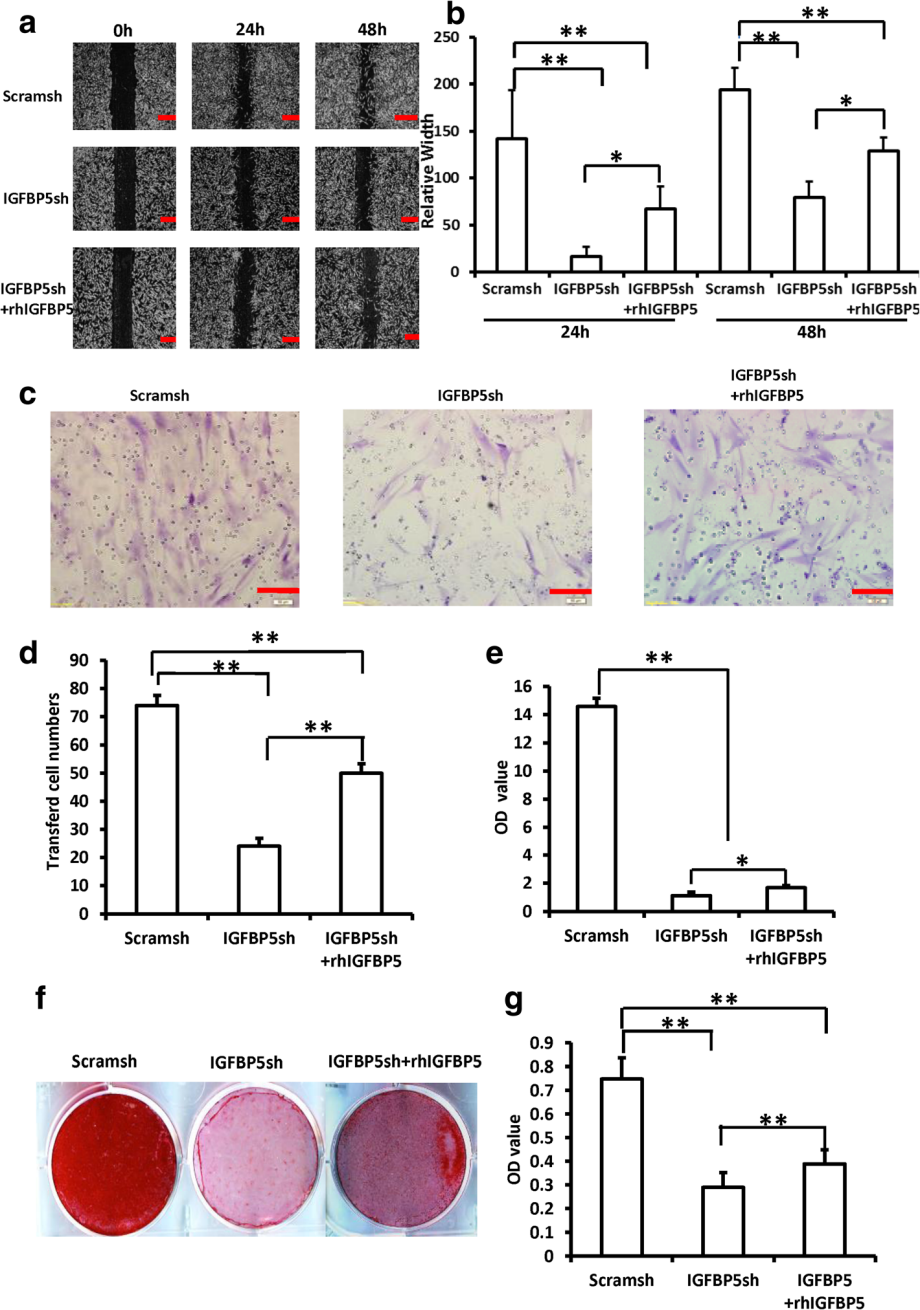
rhIGFBP5 rescued the impaired functions of *IGFBP5*-silenced-PDLSCs in inflammatory condition. TNFα (10 ng/ml) was used to treat PDLSCs. **a**, **b** The scratch-simulated migration assay results. Scale bar: 100 μm. **c**, **d** The Transwell chemotaxis assay results. Scale bar: 50 μm. **e** ALP activity. **f** Alizarin red staining. **g** Cell Counting Kit-8 assay results. One-way ANOVA was used to verify statistical significance. Error bars represent SD (n = 3). *P ≤ 0.05; **P ≤ 0.01. *rhIGFBP5* recombinant human insulin-like growth factor binding protein 5

To further verify the function of rhIGFBP5 in MSCs in the inflammatory condition, we performed rescue experiments in *IGFBP5*-silenced PDLSCs. The scratch-simulated wound migration assay results showed that 0.5 ng/ml rhIGFBP5 enhanced migration ability in *IGFBP5*-silenced PDLSCs under 10 ng/ml TNFα treatment (Fig. 3a, b). The Transwell chemotaxis assay results showed that 0.5 ng/ml rhIGFBP5 promoted chemotaxic ability in *IGFBP5*-silenced PDLSCs under TNFα treatment (Fig. 3c, d). After osteogenic induction, ALP activity assay and ARS results showed that 0.5 ng/ml rhIGFBP5 rescued the osteogenic differentiation capacity which was impaired in *IGFBP5*-silenced PDLSCs under TNFα treatment (Fig. 3e, f). Similarly, Cell Counting Kit-8 assay results confirmed that 0.5 ng/ml notably restored the impaired cell proliferation ability in *IGFBP5*-silenced PDLSCs under TNFα treatment (Fig. 3g).

### Local injection of rhIGFBP5 accelerated periodontal tissue regeneration and relieved local inflammation in a swine periodontitis model

**Fig. 4:**
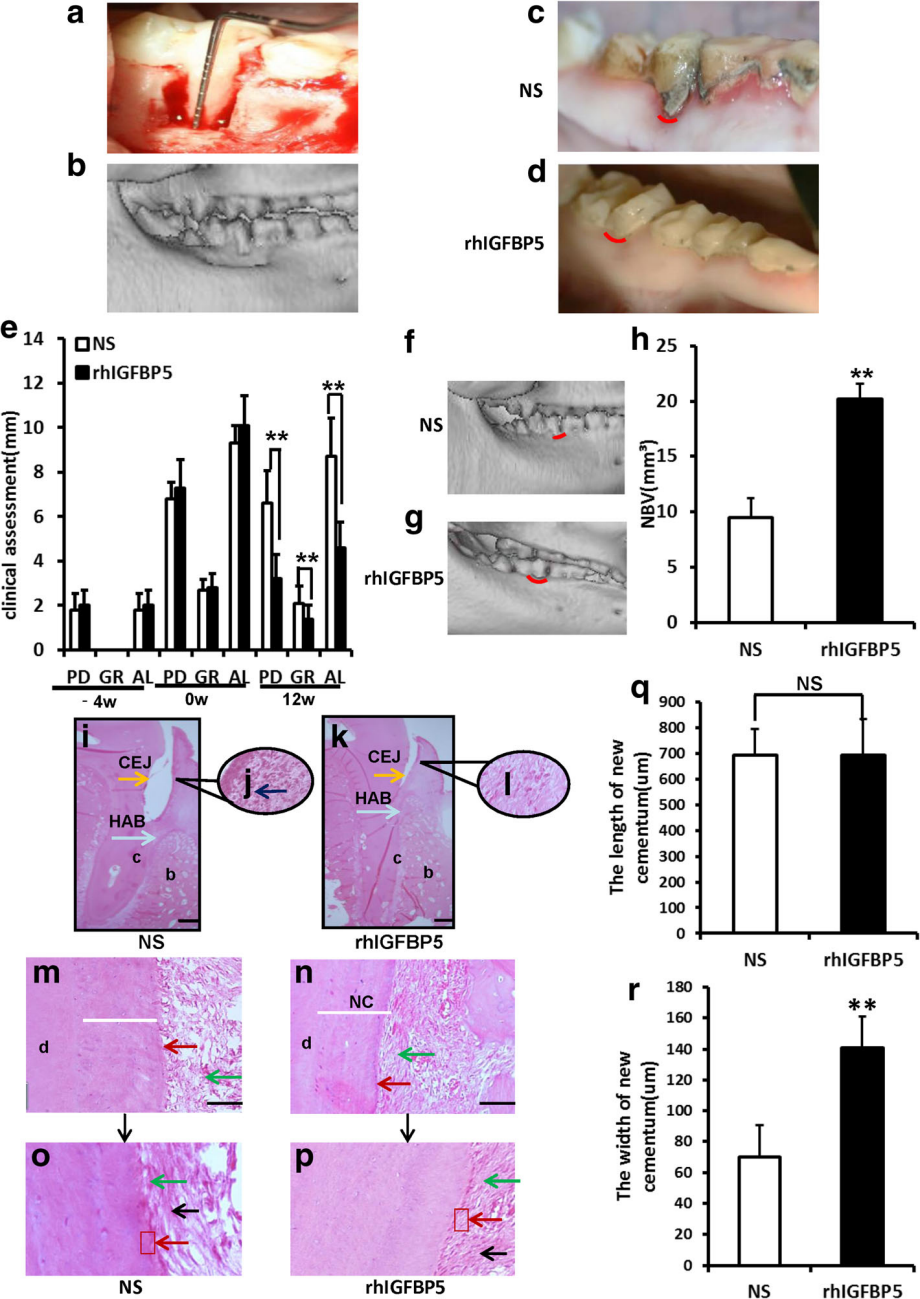
Local injection of rhIGFBP5 promoted periodontal tissue regeneration and relieved local inflammation in a swine periodontitis model. **a**, **b** A minipig model of periodontitis was established. Intraoral photographs (**a**). Three-dimensional reconstructive CT image (**b**). **c**, **d** Intraoral manifestations indicated that local injection of rhIGFBP5 alleviated local inflammation in comparison with the 0.9% NaCl group at 12 weeks after injection. **e** Clinical assessment of periodontal tissue regeneration. **f**, **g** Three-dimensional reconstructive CT scan exhibiting bone formation after injection at 12 weeks. **h** Quantitative analysis of the volume of new bone formation. **i**-**p** H&E staining exhibiting new periodontal tissue regeneration in the periodontal lesion area in the 0.9% NaCl group (**i**, **j**, **m**, **o**), and the rhIGFBP5 injection group (**k**, **l**, **n**, **p**). **m**–**p** H&E staining indicated new cementum and Sharpey’s fibers in the 0.9% NaCl group (**m**, **o**), and the rhIGFBP5 injection group (**n**, **p**). **q**, **r** Quantitative analysis of new cementum. Scale bar: 1 mm (**i**, **k**), 50 μm (**m**, **n**), 20 μm (**o**, **p**). *CEJ* cemento-enamel junction, *HAB* height of alveolar bone, *c* cementum, *b* bone, *d* dentin, *NC* new cementum, *Blue arrow*, inflammatory cells; *green arrow*, Sharpey’s fibers; *red arrow*, cementoblast; *black arrow*, PDLSCs. Student’s *t* test was implemented to test statistical significance. Error bars represent SD (*n* = 6). *NS* no significant difference. **P ≤ 0.01. *AL* attachment loss, *GR* gingival recession, *NBV* new bone volume, *PD* probing depth, *rhIGFBP5* recombinant human insulin-like growth factor binding protein 5

To further verify the effect of rhIGFBP5 on periodontal tissue regeneration in an inflammatory niche, we established a periodontitis model in minipigs (Fig. 4a, b). The areas of periodontal defects were locally injected with either a 0.9% NaCl solution or 0.5 ng/ml rhIGFBP5. At 12 weeks post-injection, the inflammation of gingival tissue was still severe in the 0.9% NaCl group, which accumulated a large amount of calculus and plaque around the marginal gingiva (Fig. 4c). However, in the rhIGFBP5 injection group, the gingival tissue was recovered and similar to the healthy gingiva (Fig. 4d). Clinical assessments of periodontal tissue regeneration were also performed. At 12 weeks post-injection, the PD was 6.6 ± 1.46 mm in the 0.9% NaCl group and 3.2 ± 1.10 mm in the rhIGFBP5 injection group. AL values were 8.7 ± 1.71 mm in the 0.9% NaCl group and 4.6 ± 1.15 mm in the rhIGFBP5 injection group. GR values were 2.1 ± 0.76 mm in the 0.9% NaCl group and 1.4 ± 0.61mm in the rhIGFBP5 injection group (Fig. 4e). These results showed that local injection of rhIGFBP5 prominently promoted periodontal tissue regeneration in comparison with the control group. In addition, three-dimensional reconstructive CT scans showed that more new alveolar bone was formed in the rhIGFBP5 injection group compared with the 0.9% NaCl group; the new alveolar bone in the rhIGFBP5 injection group exhibited approximately normal height (Fig. 4g), while the control group displayed little alveolar bone formation (Fig. 4f). Furthermore, we assessed the quantity of new alveolar bone regeneration. The results showed that the volume of new bone regeneration was 20.19 mm^3^ ± 1.37 mm^3^ in the rhIGFBP5 injection group and 9.48 mm^3^ ± 1.78 mm^3^ in the 0.9% NaCl group (Fig. 4h), indicating that the local injection of rhIGFBP5 promoted bone regeneration in the periodontal supported tissue.

Histopathological detection was also used to investigate the quantity of periodontal tissue regeneration. Histopathological photomicrographs indicated that the height of new alveolar bone and periodontal tissues were at nearly normal levels in the rhIGFBP5 injection group (Fig. 4k); much better than that in the 0.9% NaCl group (Fig. 4i). In addition, deep periodontal pockets, attachment loss and a large amount of inflammatory cells were still observed in the 0.9% NaCl group (Fig. 4i-j). Moreover, histopathological photomicrographs and the quantitative analysis results showed that the new cementum was thicker and more mature in the rhIGFBP5 injection group (Fig. 4n, r) compared to the control group (Fig. 4m, r). In addition, more cementoblasts were regenerated in the periodontal lesion areas and more new Sharpey’s fibers were observed in the rhIGFBP5 injection group (Fig. 4n, p) compared to the 0.9% NaCl group (Fig. 4m, o). However, we found no remarkable difference in the length of new cementum between the rhIGFBP5 injection group and the 0.9% NaCl group (Fig. 4q).

**Fig. 5:**
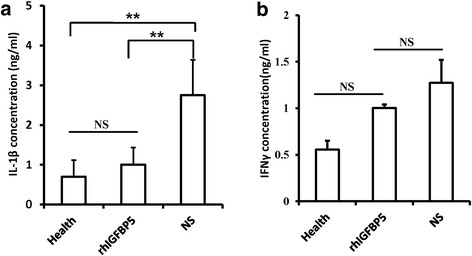
Local injection of rhIGFBP5 inhibited the expression of IL-1β in a minipig periodontitis model. **a** ELISA assay results indicated rhIGFBP5 decreased expression of IL-1β in gingival crevicular fluid. **b** The expression of IFNγ was not significantly different between the rhIGFBP5 group and the untreated group. One-way ANOVA was performed to determine statistical significance. Error bars represent SD (n = 6). *NS* no significant difference. **P ≤ 0.01. *IFNγ* interferon gamma, *IL*-*1β* interleukin 1 beta, *NS*, no significant difference. *rhIGFBP5* recombinant human insulin-like growth factor binding protein 5

To assess whether rhIGFBP5 had an anti-inflammatory function in the minipig periodontitis model, an ELISA assay was used to examine inflammatory factors, including interleukin 1 beta (IL-1β) and interferon gamma (IFNγ), in gingival sulcus fluid from the minipigs. The results revealed that the level of IL-1β was significantly decreased in the rhIGFBP5 injection group compared to the control group and almost restored to a healthy level (Fig. 5a). ELISA results showed that the expression of IFNγ was decreased in the rhIGFBP5 injection group, but there was no significant difference between these three groups (Fig. 5b).

In the end, routine blood, biochemical and immunoglobulin tests in whole blood showed no significant change between pre-injection and post-injection in the two groups (data not shown), indicating no obvious adverse effect in the minipigs that received injection of rhIGFBP5 or 0.9% NaCl.

### BCOR and KDM6B formed a protein complex which might negatively regulate the expression of *IGFBP5* by promoting histone K27 methylation in the *IGFBP5* promoter

**Fig. 6:**
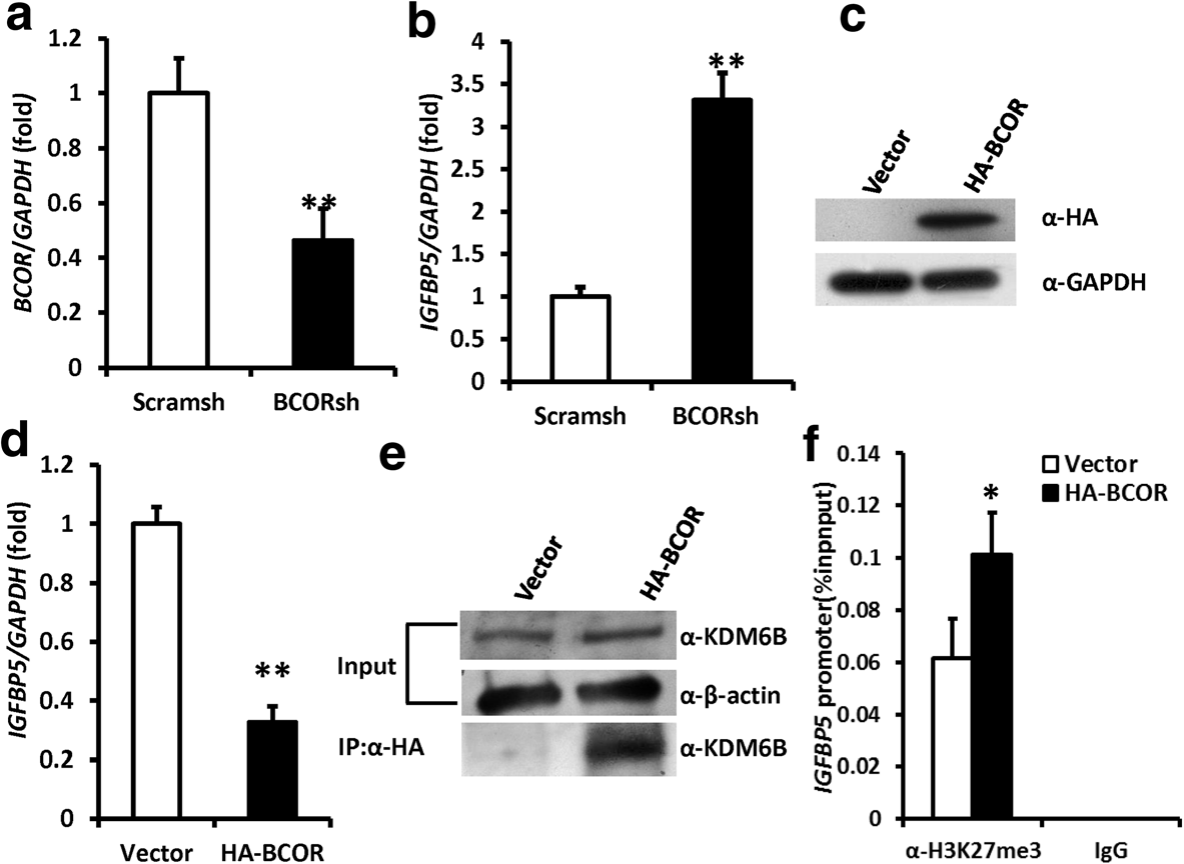
BCOR negatively regulated *IGFBP5* expression by association with KDM6B in PDLSCs. **a** Short hairpin RNAs were used to infect PDLSCs and silence *BCOR* (*BCOR*sh) or scramble shRNA (Scramsh). The knockdown of *BCOR* in PDLSCs was determined by real-time RT-PCR. *GAPDH* was utilized as an internal control. **b** The knockdown of *BCOR* promoted *IGFBP5* expression in PDLSCs as verified by real-time RT-PCR. **c** Western blot showed the overexpression of *BCOR* in PDLSCs. **d** Real-time PCR results showed that overexpression of *BCOR* decreased *IGFBP5* expression in PDLSCs. **e** Co-IP assay results showed the formation of BCOR-KDM6B protein complex in PDLSCs. β-actin was used as an internal control. **f** ChIP assay demonstrated that overexpression of *BCOR* resulted in changes in histone K27 trimethylation (H3K27me3) in the promoter of *IGFBP5*. Student’s *t* test was utilized to test statistical significance. Error bars represent SD (n = 3). *p ≤ 0.05; **p ≤ 0.01. *BCOR* BCL6 co-repressor, *GAPDH* glyceraldehyde-3-phosphate dehydrogenase, *IGFBP5* insulin-like growth factor binding protein 5, *KDM6B* lysine (K)-specific demethylase 6B

Our research group previously found that *BCOR* mutation led to increased expression of *IGFBP5*. Next, we tested the relationship of *BCOR* and *IGFBP5* in MSCs. A short hairpin RNA (shRNA) was designed to target *BCOR* and introduced into PDLSCs via lentiviral infection. After selection, the knockdown efficiency was tested by real-time RT-PCR (Fig. 6a). Real-time RT-PCR results revealed that *BCOR* knockdown promoted *IGFBP5* expression in PDLSCs (Fig. 6b). The *HA*-*BCOR* sequence was constructed in a retroviral vector and then transduced into PDLSCs by retroviral infection (Fig. 6c). *BCOR* overexpression suppressed *IGFBP5* expression as measured by real-time RT-PCR analysis (Fig. 6d). A Co-IP assay confirmed that BCOR and KDM6B combined together to form a protein complex in PDLSCs (Fig. 6e).

Next, a ChIP assay was designed to detect whether *BCOR* altered methylation of histone K27 on the promoter of *IGFBP5*. The results showed that HA-BCOR raised the level of H3K27me3 in the *IGFBP5* promoter (Fig. 6f), indicating that HA-BCOR repressed *IGFBP5* transcription by increasing histone K27 methylation in the promoter of *IGFBP5*.

## Discussion

Improving endogenous MSCs populations and their functions is a key factor in restoring periodontal tissues in periodontitis, especially in the situation without exogenous MSCs application [17, 28]. Application of simple growth factors to recruit and activate the endogenous MSCs is an alternative method. Our previous study revealed that IGFBP5 expression was decreased in PDLSCs and local tissues in periodontitis patients, and the lost expression of IGFBP5 in PDLSCs impaired the osteogenic differentiation potential, which suggested that maintaining IGFBP5 expression might be beneficial for tissue regeneration and inflammation control in periodontitis. Indeed, we found that combined usage of IGFBP5 and exogenous MSCs could promote periodontal tissue regeneration and alleviate local inflammation [24]. Thus, in this study, we investigated the possibility of IGFBP5 protein application as a method of treatment for periodontitis independent of exogenous MSCs. First, using TNFα to mimic an inflammatory niche, we discovered that the depletion of *IGFBP5* inhibited the migration, chemotaxis, osteogenic differentiation and cell proliferation ability of PDLSCs in the inflammatory condition. These discoveries further confirmed that loss of IGFBP5 impaired MSC function in periodontitis. Furthermore, we confirmed that 0.5 ng/ml rhIGFBP5 enhanced the migration, chemotaxis, osteogenic differentiation and cell proliferation potentials of PDLSCs and rescued the impaired functions of *IGFBP5*-silenced-PDLSCs in the inflammatory condition. In addition to PDLSCs, BMSCs in alveolar bone are also an alternative endogenous cell resource for periodontitis treatment. Thus, we elucidated that rhIGFBP5 also promoted the migration, chemotaxis, osteogenic differentiation and cell proliferation potentials of BMSCs in the inflammatory condition. Our results indicated that rhIGFBP5 could not only recruit the endogenous MSCs participating in tissue restoration but also promote the proliferation and differentiation capacities of MSCs in the inflammatory niche, suggesting that rhIGFBP5 might be helpful in rescuing the impaired functions of endogenous MSCs.

To further evaluate the effect of rhIGFBP5 on periodontal tissue regeneration, we created a preclinical model of periodontitis by constructing bone defects in minipigs. According to clinical observation, three-dimensional reconstructive CT scans and histopathological photomicrographs, we found that local injection of rhIGFBP5 notably promoted alveolar bone regeneration. Histopathological photomicrographs of the minipig model indicated that local injection of rhIGFBP5 increased periodontal tissues, including new thicker cementum and periodontal ligament which were regenerated in the lesion area, where newly-shaped Sharpey’s fibers inserted into regenerated cementum. Moreover, fewer inflammatory cells had infiltrated the gingival tissue of the defect areas in the rhIGFBP5 treatment group.

A series of intricate inflammatory and immune responses are involved in the progression of periodontal inflammation, and it has been realized that the expression of cytokines reflects T-helper activities, which results in periodontal disease [29, 30]. High levels of IL-1β, and IFNγ, which play key roles in inflammatory immune responses, have been identified in periodontal disease. IL-1β is an inflammatory mediator produced by macrophages and dendritic cells, but is also secreted by PDLSCs, gingival fibroblasts and osteoblasts. Moreover, IL-1β is increased in the saliva and gingival sulcus fluid of periodontitis patients and induced bone destruction [31–34]. IFNγ is secreted by Th1 cells and can activate cell-mediated immunity. Furthermore, IFNγ can inhibit MSC-mediated bone formation and induce MSC apoptosis via immune T-cell-based regulatory mechanisms [35]. In our study, we discovered that local injection of rhIGFBP5 significantly decreased IL-1β expression, indicating that rhIGFBP5 could regulate inflammatory immune responses in periodontitis. Although there was no significant difference of IFNγ, the ELISA results still showed that the expression of IFNγ was decreased following rhIGFBP5 injection. In a future study, we should focus on identifying the inflammatory immune responses related to susceptibility for periodontal disease progression in periodontitis and adjust the applied method of rhIGFBP5, such as dose, time and release method, to obtain the ideal effect. Taken together, our results demonstrated that rhIGFBP5 can restore the tissue defects resulting from periodontitis and has inflammatory immune regulatory effects in a minipig model.

In addition to local application of IGFBP5 protein, we also found some agents that regulate the expression of IGFBP5 for control of endogenous MSC function, and thus, the regulation mechanism of IGFBP5 in MSCs should be elucidated. Our previous findings showed that *BCOR* mutation increases *IGFBP5* expression, and *BCOR* mutation enhances the osteo/dentinogenic functions of MSCs [36]. In the present study, we confirmed that *BCOR* could negatively regulate *IGFBP5* expression in PDLSCs. Additionally, previous experiments verified that depletion of *KDM6B* promotes methylation of H3K27me3 on the *IGFBP5* promoter, and controls the transcription of *IGFBP5* in MSCs [24, 37]. Therefore, we wondered whether BCOR regulated *IGFBP5* expression by associating with KDM6B. A Co-IP assay was used to reveal the combination of BCOR and KDM6B, and the results showed that BCOR and KDM6B could combine together to form a complex, indicating that BCOR might associate with KDM6B to regulate *IGFBP5* expression in MSCs. In addition, ChIP assay results confirmed that overexpression of *BCOR* enhanced H3K27me3 methylation on the promoter of *IGFBP5*. These results suggested that BCOR-KDM6B might form a protein complex regulating the expression of *IGFBP5* by controlling H3K27me3 methylation on the *IGFBP5* promoter. However, further investigations will be needed to illuminate the precise regulation mechanism of *IGFBP5*.

## Conclusions

In summary, our discovery reveals that rhIGFBP5 can activate the functions of MSCs in an inflammatory niche and that BCOR can form a protein complex with histone demethylase KDM6B, thus regulating *IGFBP5* transcriptionally by regulating H3K27me3 methylation on the *IGFBP5* promoter. Our findings provide insight into the mechanism underlying the activated capacities of MSCs, identified IGFBP5 as a potential cytokine for improving tissue regeneration and periodontitis treatment independent of exogenous MSCs, and suggest that IGFBP5 has potential application in the dental clinic.

## Additional files

Additional file 1: Table S1. Primer sequences used in the real-time RT-PCR. (DOC 32 kb)

Additional file 2: Figure S1. rhIGFBP5 enhanced the functions of BMSCs in the inflammatory condition. BMSCs were treated with 10 ng/ml TNFα. **a**, **b** The scratch-simulated wound migration assay results indicated that 0.5 ng/ml rhIGFBP5 promoted migration ability in BMSCs. Scale bar: 100 μm. **c**, **d** The Transwell chemotaxis assay results showed that 0.5 ng/ml rhIGFBP5 promoted chemotaxis ability in BMSCs. Scale bar: 50 μm. **e** ALP activity assay showed that 0.5 ng/ml rhIGFBP5 enhanced ALP activity. **f** 0.5 ng/ml rhIGFBP5 promoted mineralization in BMSCs, as verified by Alizarin red staining. **g** Cell counting kit-8 assay results showed that 0.5 ng/ml rhIGFBP5 accelerated cell proliferation in BMSCs. Student’s *t* test was implemented to detect statistical significance. Error bars represent SD (n = 3). *P ≤ 0.05; **P ≤ 0.01. (TIFF 3755 kb)

## Abbreviations

AL: Attachment loss
ALP: Alkaline phosphatase
ARS: Alizarin red staining
BCOR: BCL6 co-repressor
BMSCs: Bone marrow stromal stem cells
Co-IP: Co-immunoprecipitation
CT: Computed tomography
EDTA: Ethylene diamine tetraacetic acid
GAPDH: Glyceraldehyde-3-phosphate dehydrogenase
GR: Gingival recession
H3K27me3: Histone H3K27 trimethylation
IFNγ: Interferon gamma
IGFBP5: Insulin-like growth factor binding protein 5
IL-1β: Interleukin 1 beta
KDM6B: Lysine (K)-specific demethylase 6B
MSCs: Mesenchymal stem cells
NBV: New bone volume
PD: Probing depth
PDLSCs: Periodontal ligament stem cells
rhIGFBP5: Recombinant human insulin-like growth factor binding protein 5
TNFα: Tumor necrosis factor alpha
