# Supplementary material for: Local application of IGFBP5 protein enhanced periodontal tissue regeneration via increasing the migration, cell proliferation and osteo/dentinogenic differentiation of mesenchymal stem cells in an inflammatory niche
Source: Stem Cell Res Ther. 2017 Sep 29;8:210. doi: 10.1186/s13287-017-0663-6 (PMC5622495; doi:10.1186/s13287-017-0663-6)
Supplement: Supplementary file 1 — Primer sequences used in the real-time RT-PCR. (DOC 32 kb) [file 13287_2017_663_MOESM1_ESM.doc]

**Additional file 1: Table S1. Primers sequences used in the real-time RT-PCR**

**Gene Symbol Primer Sequences (5’-3’)**

*GAPDH*-F CGGACCAATACGACCAAATCCG

*GAPDH*-R AGCCACATCGCTCAGACACC

*IGFBP5*-F GCACCTGAGATGAGACAGGA

*IGFBP5*-R TGTAGAATCCTTTGCGGTCA

*BCOR*-F CATAGTGCTTGTGGAACTCCG

*BCOR*-R GGACACAGCTCTCCTGTTGC
