# Supplementary figures and images for: Local application of IGFBP5 protein enhanced periodontal tissue regeneration via increasing the migration, cell proliferation and osteo/dentinogenic differentiation of mesenchymal stem cells in an inflammatory niche
Source: Stem Cell Res Ther. 2017 Sep 29;8:210. doi: 10.1186/s13287-017-0663-6 (PMC5622495; doi:10.1186/s13287-017-0663-6)

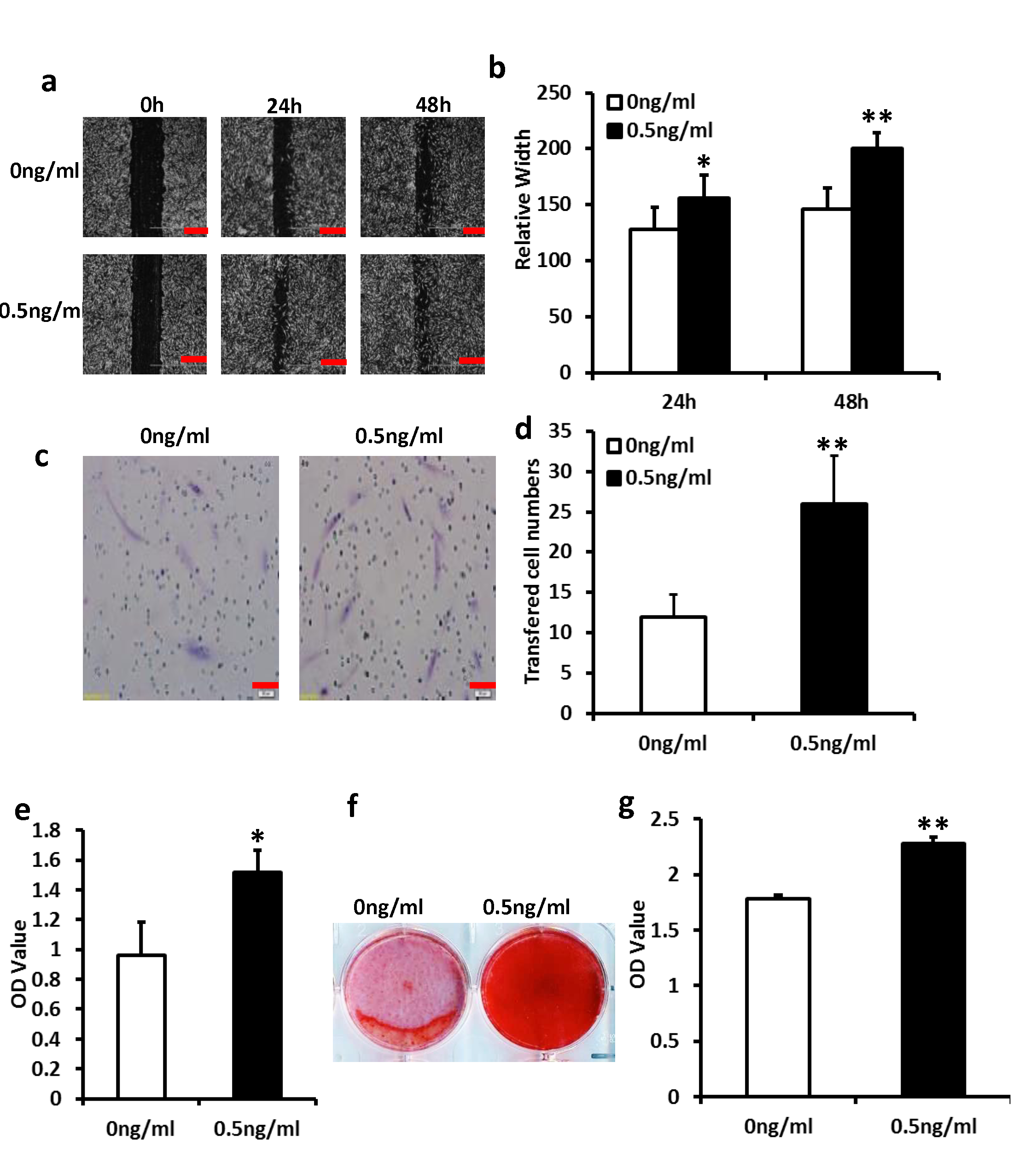

Supplement: Supplementary file 2 — rhIGFBP5 enhanced the functions of BMSCs in the inflammatory condition. BMSCs were treated with 10 ng/ml TNFα. a, b The scratch-simulated wound migration assay results indicated that 0.5 ng/ml rhIGFBP5 promoted migration ability in BMSCs. Scale bar: 100 μm. c, d The Transwell chemotaxis assay results showed that 0.5 ng/ml rhIGFBP5 promoted chemotaxis ability in BMSCs. Scale bar: 50 μm. e ALP activity assay showed that 0.5 ng/ml rhIGFBP5 enhanced ALP activity. f 0.5 ng/ml rhIGFBP5 promoted mineralization in BMSCs, as verified by Alizarin red staining. g Cell counting kit-8 assay results showed that 0.5 ng/ml rhIGFBP5 accelerated cell proliferation in BMSCs. Student’s t test was implemented to detect statistical significance. Error bars represent SD (n = 3). *P ≤ 0.05; **P ≤ 0.01. (TIFF 3755 kb) [file 13287_2017_663_MOESM2_ESM.tiff]
